# Interferon-γ Activates Nuclear Factor-κ B in Oligodendrocytes through a Process Mediated by the Unfolded Protein Response

**DOI:** 10.1371/journal.pone.0036408

**Published:** 2012-05-04

**Authors:** Yifeng Lin, Stephanie Jamison, Wensheng Lin

**Affiliations:** 1 Department of Cell Biology and Neuroscience, University of South Alabama College of Medicine, Mobile, Alabama, United States of America; 2 Department of Comparative Medicine, University of South Alabama College of Medicine, Mobile, Alabama, United States of America; University of Pecs Medical School, Hungary

## Abstract

Our previous studies have demonstrated that the effects of the immune cytokine interferon-**γ** (IFN-γ) in immune-mediated demyelinating diseases are mediated, at least in part, by the unfolded protein response (UPR) in oligodendrocytes. Data indicate that some biological effects of IFN-γ are elicited through activation of the transcription factor nuclear factor-κB (NF-κB). Interestingly, it has been shown that activation of the pancreatic endoplasmic reticulum kinase (PERK) branch of the UPR triggers NF-κB activation. In this study, we showed that IFN-γ-induced NF-κB activation was associated with activation of PERK signaling in the oligodendroglial cell line Oli-neu. We further demonstrated that blockage of PERK signaling diminished IFN-γ-induced NF-κB activation in Oli-neu cells. Importantly, we showed that NF-κB activation in oligodendrocytes correlated with activation of PERK signaling in transgenic mice that ectopically express IFN-γ in the central nervous system (CNS), and that enhancing IFN-γ-induced activation of PERK signaling further increased NF-κB activation in oligodendrocytes. Additionally, we showed that suppression of the NF-κB pathway rendered Oli-neu cells susceptible to the cytotoxicity of IFN-γ, reactive oxygen species, and reactive nitrogen species. Our results indicate that the UPR is involved in IFN-γ-induced NF-κB activation in oligodendrocytes and suggest that NF-κB activation by IFN-γ represents one mechanism by which IFN-γ exerts its effects on oligodendrocytes in immune-mediated demyelinating diseases.

## Introduction

The immune cytokine interferon-γ (IFN-γ) plays a critical role in immune-mediated demyelinating diseases multiple sclerosis (MS) and experimental autoimmune encephalomyelitis (EAE) [1], [2]. Recent studies suggest that the actions of IFN-γ in MS and EAE are mediated, at least in part, by its effects on oligodendrocytes [3], [4], [5]. Nevertheless, the molecular mechanisms by which IFN-γ influences the function and viability of oligodendrocytes remain elusive. The transcription factor nuclear factor-κB (NF-κB) is a hetero- or homodimer of the Rel family of proteins, including p65, c-Rel, RelB, p50, and p52 [6], [7]. In the quiescent state, NF-κB remains inactive in the cytoplasm through interaction with inhibitory proteins, NF-κB inhibitors (IκBs). Activation of NF-κB involves the cytoplasmic degradation of IκBs, allowing the translocation of NF-κB into the nucleus where the dimer binds to the κB consensus DNA sequence and regulates transcription of genes that are essential for innate and adaptive immunity and for regulation of cell apoptosis and survival. There is evidence that the NF-κB pathway is involved in the pathogenesis of MS and EAE [7], [8], [9]. Activation of the NF-κB pathway has been observed in oligodendrocytes in these diseases [8]. Importantly, several lines of evidence have suggested that the NF-κB pathway is involved in mediating the actions of IFN-γ [10], [11]. Therefore, it is interesting to determine the involvement of the NF-κB pathway in the effects of IFN-γ on oligodendrocytes.

While evidence is accumulating that IFN-γ activates the NF-κB pathway [11], [12], its underlying mechanisms remain elusive. Endoplasmic reticulum (ER) stress, initiated by the accumulation of unfolded or misfolded proteins in the ER lumen, activates an adaptive program known as the unfolded protein response (UPR) [13], [14]. In eukaryotic cells, monitoring of the ER lumen and signaling through the canonical branches of the UPR are mediated by three ER-resident transmembrane proteins, pancreatic ER kinase (PERK), inositol requiring enzyme 1 (IRE1), and activating transcription factor 6 (ATF6). PERK activation inhibits global protein translation, but stimulates the expression of certain stress-induced cytoprotective genes by phosphorylating translation initiation factor 2α (eIF2α). Interestingly, recent discoveries have demonstrated that activation of PERK signaling triggers NF-κB activation by repression of IκBα translation [15], [16]. Our previous studies have shown that IFN-γ activates PERK signaling in oligodendrocytes in immune-mediated demyelinating diseases [3], [17], [18]. Thus we examine whether IFN-γ activates the NF-κB pathway in oligodendrocytes by a process mediated by the PERK branch of the UPR.

In this study, we show that IFN-γ activates both the NF-κB pathway and the PERK pathway in the oligodendroglial cell line Oli-neu. We also show that suppression of the NF-κB pathway makes Oli-neu cells susceptible to the cytotoxicity of IFN-γ, reactive oxygen species, and reactive nitrogen species. Moreover, we demonstrate that blockage of PERK signaling diminishes NF-κB activation in Oli-neu cells in response to IFN-γ. Importantly, we provide evidence that PERK signaling contributes to IFN-γ-induced NF-κB activation in oligodendrocytes in transgenic mice that ectopically express IFN-γ in the CNS. Collectively, this study reveals a novel mechanism responsible for IFN-γ-induced NF-κB activation and suggests that the NF-κB pathway is involved in modulating the response of oligodendrocytes to IFN-γ in immune-mediated demyelinating diseases.

## Materials and Methods

### Cell Culture

The Oli-neu cell line [19] was a generous gift from Dr. Brian Popko (University of Chicago, Chicago, Illinois), which was maintained in Dulbecco’s Modified Eagle Medium (DMEM; Invitrogen, Carlsbad, California) supplemented with 100 µg/ml apotransferrin (Sigma-Aldrich, St. Louis, Missouri), 30 nM sodium selenite (Sigma-Aldrich), 5 µg/ml insulin (Sigma-Aldrich), 100 µM putrescine (Sigma-Aldrich), 20 nM progesterone (Sigma-Aldrich), 2 mM L-glutamine (Invitrogen), 25 mM HEPES (Invitrogen), 10 ng/ml Biotin (Sigma-Aldrich), 1 mM sodium Pyruvate (Invitrogen), 1% penicillin/streptomycin (Invitrogen), and 1% horse serum (Invitrogen). To suppress the activity of the NF-κB pathway, Oli-neu cells were transfected with a mammalian expression plasmid pcDNA3.1-IκBαΔN that contains the hygromycin resistance gene using Lipofectamine 2000 transfection reagent (Invitrogen) according to the manufacturer’s instructions. The IκBαΔN cDNA clone was a generous gift from Dr. Dean W. Ballard (Vanderbilt University, Nashville, Tennessee). The stably transfected cells were selected with 200 µg/ml hygromycin. To block the activity of the PERK pathway, Oli-neu cells were transfected with a mammalian expression plasmid pBabe-PERKΔC that contains the puromycin resistance gene [20] using Lipofectamine 2000 transfection reagent (Invitrogen) according to the manufacturer’s instructions. The stably transfected cells were selected with 0.5 µg/ml puromycin.

### MTT Assay, BrdU Cell Proliferation Assay, and Caspase-3 Activity Assay

Oli-neu cells were plated 1×10^4^ per well in 96-well microtiter plates. After 24 hrs, the cells were treated with either IFN-γ (EMD Biosciences, San Diego, California), hydrogen peroxide (H_2_O_2_; Sigma-Aldrich), or a nitric oxide (NO) donor sodium nitroprusside (SNP; Sigma-Aldrich) for 24 hrs. Cell viability is determined by the MTT Assay Kit (Promega, Madison, Wisconsin) according to the manufacturer’s instructions.

Oli-neu cells were plated at 5×10^3^ per well in 96-well microtiter plates. After 24 hrs, IFN-γ and Bromodeoxyuridine (BrdU) labeling solution (Millipore, Billerica, Massachusetts) were added to the culture media for 24 hrs. Cell proliferation was determined using the Colorimetric BrdU Cell Proliferation kit (Millipore) according to the manufacturer’s instructions.

Oli-neu cells were treated with IFN-γ for 24 hrs. The activity of caspase-3 was assessed using the Colorimetric Caspase-3 Assay Kit (Sigma-Aldrich) according to the manufacturer’s instructions.

### NF-κB Electrophoretic Mobility Shift Assay (EMSA)

Oli-neu cells treated with IFN-γ (EMD Biosciences) for 16 hrs were rinsed with ice-cold PBS. Nuclear extract from cells was prepared using NE-PER Nuclear and Cytoplasmic Extraction Kit (Pierce, Rockford, Illinois) plus complete Protease Inhibitor Cocktail Kit (Roche, Indianapolis, Indiana) following the manufacturers’ instructions and stored at −80°C until used. The protein content of each extract was determined by DC Protein Assay (Bio-Rad Laboratories, Hercules, California). Complementary NF-κB consensus oligonucleotides 5′-AGTTGAGGGGACTTTCCCAGGC-3′ and 5′-GCCTGGGAAAGTCCCCTCAACT-3′ (Integrated DNA Technologies, Coralville, Iowa) were end-labeled with biotin separately using the biotin 3′-end DNA Labeling Kit (Pierce) and then annealed by heating to 95°C for 5 min followed by slow cooling to room temperature. Probes were stored at –20°C until used. EMSA was performed with the LightShift Chemiluminescent EMSA Kit (Pierce) according to the manufacturer’s instructions. Briefly, nuclear extract (5 µg) was incubated with 20 fmol biotin 3′ end-labeled oligonucleotides. After electrophoresis, transfer, and cross-linking, the signal was detected by a peroxidase/luminol system. To confirm the specificity, a 200-fold excess amount of nonlabeled oligonucleotides (cold probe) was added. The density of NF-κB DNA-binding complex was quantified with Kodak 1D Image Analysis software (Kodak, Hercules, New Haven, Connecticut).

### Real-time PCR

RNA was isolated from Oli-neu cells treated with 100 U/ml IFN-γ (EMD Biosciences) for 24 hrs using TRIzol reagent (Invitrogen) and was treated with DNaseI (Invitrogen) to eliminate genomic DNA. Reverse transcription was performed using the iScript cDNA Synthesis Kit (Bio-Rad Laboratories, Hercules, California). Real-time PCR was performed with iQ Supermix (Bio-Rad Laboratories) on a real-time PCR detection system (model iQ; Bio-Rad Laboratories). The following primers and probes (Integrated DNA Technologies, Inc.) for real-time PCR were used: glyceraldehyde phosphate dehydrogenase (GAPDH) sense primer (CTCAACTACATGGTCTACATGTTCCA); GAPDH antisense primer (CCATTCTCGGCCTTGACTGT); GAPDH probe (TGACTCCACTCACGGCAAATTCAACG); CAATT enhancer binding protein homologous protein (CHOP) sense primer (CCACCACACCTGAAAGCAGAA); CHOP antisense primer (AGGTGCCCCCAATTTCATCT); CHOP probe (TGAGTCCCTGCCTTTCACCTTGGAGA); binding immunoglobulin protein (BIP) sense primer (ACTCCGGCGTGAGGTAGAAA); BIP antisense primer (AGAGCGGAACAGGTCCATGT); BIP probe (TTCTCAGAGACCCTTACTCGGGCCAAATT).

### Western Blot Analysis

Oli-neu cells treated with IFN-γ (EMD Biosciences) were rinsed with ice-cold PBS and were immediately homogenized in 5 volume of Triton X-100 buffer as previously described [17]. After incubating on ice for 15 min, the extracts were cleared by centrifugation at 14,000 rpm twice for 30 min each. The protein content of each extract was determined by DC Protein Assay (Bio-Rad Laboratories). The extracts (40 µg) were separated by SDS-PAGE and were transferred to nitrocellulose. The blots were incubated with primary antibody (see below), and the signal was revealed by chemiluminescence after reacting with HRP-conjugated second antibody. The following primary antibodies were used: phosphorylated PERK (p-PERK, 1∶1000; Santa Cruz Biotechnology, Santa Cruz, California); eIF-2α (1∶1000; Santa Cruz Biotechnology); phosphorylated eIF-2α (p-eIF-2α,1∶1000; Cell Signaling Technology, Beverly, Massachusetts); ATF4 (1∶4000; Abcam, Cambridge, Massachusetts); IκBα (1∶1000; Santa Cruz Biotechnology), c-Myc [20], and β-actin (1∶5000; Sigma-Aldrich). The density of immunoblotting was quantified with Kodak 1D Image Analysis software (Kodak, Hercules, New Haven, Connecticut).

### Mice Breeding

Line110 *GFAP/tTA* mice and line184 *TRE/IFN-γ* mice on the C57BL/6 background were mated with *growth arrest and DNA damage 34* (*GADD34*) mutant mice on the C57BL/6 background, and the resulting progeny were intercrossed to obtain *GFAP/tTA*; *TRE/IFN*-*γ*; *GADD34* WT mice and *GFAP/tTA*; *TRE/IFN*-*γ*; *GADD34* mutant mice as described in our previous papers [21], [22]. To prevent transcriptional activation of the *TRE/IFN-γ* transgene by tTA, 0.05 mg/ml doxycycline was added to the drinking water and was provided *ad libitum.* All animal procedures were conducted in complete compliance with the National Institutes of Health’s (NIH) Guide for the Care and Use of Laboratory Animals and were approved by the Institutional Animal Care and Use Committee of the University of South Alabama.

### Immunohistochemistry (IHC)

Anesthetized mice were perfused through the left cardiac ventricle with 4% paraformaldehyde in 0.1 M PBS. The brains were removed, postfixed with paraformaldehyde, cryopreserved in 30% sucrose, embedded in optimal cutting temperature compound and frozen on dry ice. Frozen sections were cut in a cryostat at a thickness of 10 µm and were treated with −20°C acetone prior to IHC. For IHC, Oli-neu cells treated with IFN-γ were fixed with 4% paraformaldehyde in 0.1 M PBS. Then the brain sections and the cells were blocked with PBS containing 10% goat serum and 0.1% Triton X-100 and incubated overnight with the primary antibody diluted in blocking solution. Appropriate fluorochrome-labeled secondary antibodies (Vector Laboratories, Burlingame, California) were used for detection. An antibody against 2′,3′-cyclic nucleotide phosphodiesterase (CNP, 1∶200; Santa Cruz Biotechnology) was used as a marker for myelin and oligodendrocytes. Antibody against active caspase-3 (1∶100; Cell Signaling Technology) was used to detect activation of caspase-3. Antibodies against p65 (1∶400, Santa Cruz Biotechnology) and the active form of p65 (1∶50, Millipore, Billerica, Massachusetts) were used to detect nucleus translocation of p65 and activation of p65, respectively. Fluorescent stained sections and cells were mounted with Vectashield mounting medium with DAPI (Vector Laboratories). The stained sections and cells were visualized with the Nikon A1 confocal microscope or the Nikon Eclipse TE2000U fluorescence inverted microscope and analyzed as previously described [17]. Briefly, quantitative p65 nucleus translocation analyses were performed in 4 separate cell preparations for each group. Each individual Oli-neu cell was imaged using the Nikon A1 confocal microscope. We randomly imaged at least 30 cells in each cell preparation. The results were presented as the percentage of cells with p65 nucleus translocation.

### Statistics

Data are expressed as mean ± standard deviation. Multiple comparisons were statistically evaluated by one way ANOVA test using Sigmastat 3.1 software (Hearne Scientific Software). Differences were considered statistically significant if *p*<0.05.

## Results

### NF-κB Activation Prevented Oli-neu Cells from the Cytotoxicity of IFN-γ

**Figure 1:**
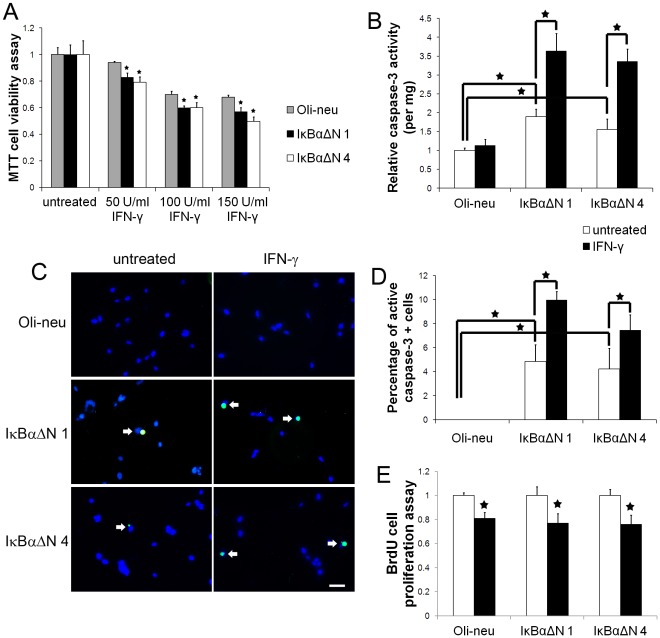
NF-κB activation promoted Oli-neu cell survival in response to IFN-γ. **A.** The cells were treated with 50 U/ml, 100 U/ml, or 150 U/ml IFN-γ for 24 hrs. MTT assay showed that IFN-γ treatment reduced the number of Oli-neu cells in a dose-dependent manner and that enforced expression of IκBαΔN further reduced the cell numbers. **B.** The cells were treated with 100 U/ml IFN-γ for 24 hrs. Caspase-3 activity assay showed that IFN-γ treatment did not alter the activity of caspase-3 in Oli-neu cells. Nevertheless, IFN-γ treatment significantly increased the activity of caspase-3 in IκBαΔN 1 and 4 cells. **C**, **D.** The cells were treated with 100 U/ml IFN-γ for 24 hrs. Active caspase-3 immunostaining showed that IFN-γ treatment did not change the percentage of active caspase-3 positive cells in Oli-neu cells. Nevertheless, IFN-γ treatment significantly increased the percentage of active caspase-3 positive cells in IκBαΔN 1 and 4 cells. **E.** BrdU cell proliferation assay showed that the treatment with 100 U/ml IFN-γ for 24 hrs significantly suppressed Oli-neu cell proliferation. Nevertheless, suppression of the NF-κB pathway did not significantly alter the sensitivity of Oli-neu cells to the antiproliferative effects of IFN-γ. The experiments were repeated at least three times, error bars represent standard deviation, asterisk *p*<0.05, scale bar = 100 µm.

Several lines of evidence have suggested that the effects of IFN-γ on oligodendroglial lineage cells are dependent on the differentiation stages of the cells [5]. It has been shown that IFN-γ is not detrimental to mature oligodendrocytes [3], [4], [17]. In contrast, several studies have shown that IFN-γ induces myelinating oligodendrocyte apoptosis [17]. Recent reports have shown that IFN-γ influences the differentiation and proliferation of oligodendrocyte precursors, but does not affect the cell viability [23], [24]. The oligodendroglial cell line Oli-neu was generated by immortalization of mitotic oligodendrocyte precursor cells with retroviral vectors containing the t-neu oncogene. Oli-neu cells adhere to and ensheath axons *in vitro* and *in vivo* in an oligodendroglial specific manner [19]. To evaluate the effects of IFN-γ on Oli-neu cells, the cells were treated with 50 U/ml, 100 U/ml, or 150 U/ml IFN-γ for 24 hrs. MTT cell viability and proliferation assay showed that IFN-γ treatment reduced the number of Oli-neu cells in a dose-dependent manner (Figure 1A). Because MTT assay is not capable of distinguishing whether the reduction of the number of Oli-neu cells is due to cell apoptosis or decreased cell proliferation, we further performed caspase-3 activity assay to determine whether IFN-γ treatment caused cell apoptosis. Interestingly, the activity of caspase-3 in Oli-neu cells that were treated with 100 U/ml IFN-γ for 24 hrs was comparable to the untreated Oli-neu cells (Figure 1B). Moreover, active caspase-3 and DAPI double labeling showed that the treatment with 100 U/ml IFN-γ did not significantly increase the number of apoptotic Oli-neu cells (Figure 1C, D). Interestingly, BrdU cell proliferation assay showed that the treatment with 100 U/ml IFN-γ significantly inhibited Oli-neu cell proliferation (Figure 1E). Taken together, these data indicate that IFN-γ suppresses Oli-neu cell proliferation but does not affect its viability.

**Figure 2:**
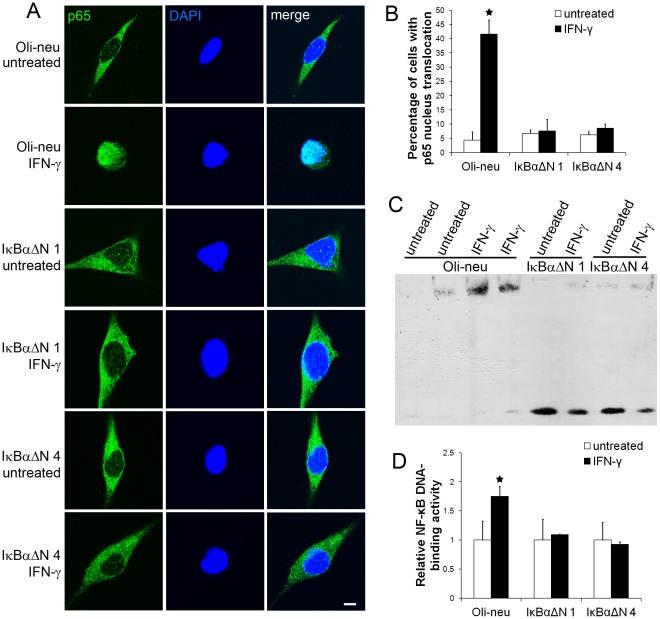
Enforced expression of IκBαΔN blocked IFN-γ-induced NF-κB activation. **A.** The cells were treated with 100 U/ml IFN-γ for 16 hrs. p65 and DAPI double labeling and confocal imaging analysis showed that p65 remained in the cytoplasm in the untreated Oli-neu cells and that IFN-γ treatment induced translocation of p65 from the cytoplasm to the nucleus in Oli-neu cells. Interestingly, IFN-γ treatment did not result in p65 nucleus translocation in IκBαΔN 1 and 4 cells. **B.** Quantitative analysis showed that the percentage of Oli-neu cells with p65 nucleus translocation was significantly increased after 16 hrs of 100 U/ml IFN-γ treatment. Nevertheless, enforced expression of IκBαΔN blocked IFN-γ-induced p65 nucleus translocation in IκBαΔN 1 and 4 cells. **C,** **D.** The cells were treated with 100 U/ml IFN-γ for 16 hrs. EMSA analysis showed a significant increase in NF-κB DNA-binding activity in Oli-neu cells treated with IFN-γ. Nevertheless, enforced expression of IκBαΔN blocked IFN-γ-induced increase in NF-κB DNA-binding activity in IκBαΔN 1 and 4 cells. The experiments were repeated at least three times, error bars represent standard deviation, asterisk *p*<0.05, scale bar = 20 µm.

It has been shown that the NF-κB pathway is involved in mediating the actions of IFN-γ in the cells [10], [11]. Therefore, it is interesting to determine the potential role of the NF-κB pathway in the effects of IFN-γ on Oli-neu cells. p65 and DAPI double labeling and confocal imaging analysis showed that p65 remained in the cytoplasm in the majority of the untreated Oli-neu cells (Figure 2A). Interestingly, we found that IFN-γ treatment induced translocation of p65 from the cytoplasm to the nucleus in Oli-neu cells, which indicates NF-κB activation. Quantitative analysis showed that the percentage of Oli-neu cells with p65 nucleus translocation was significantly increased after 16 hrs of 100 U/ml IFN-γ treatment (Figure 2B). Moreover, EMSA analysis showed a significant increase in NF-κB DNA-binding activity in Oli-neu cells treated with 100 U/ml IFN-γ for 16 hrs (Figure 2C, D). Thus, these data demonstrate that IFN-γ activates the NF-κB pathway in Oli-neu cells.

**Figure 3:**
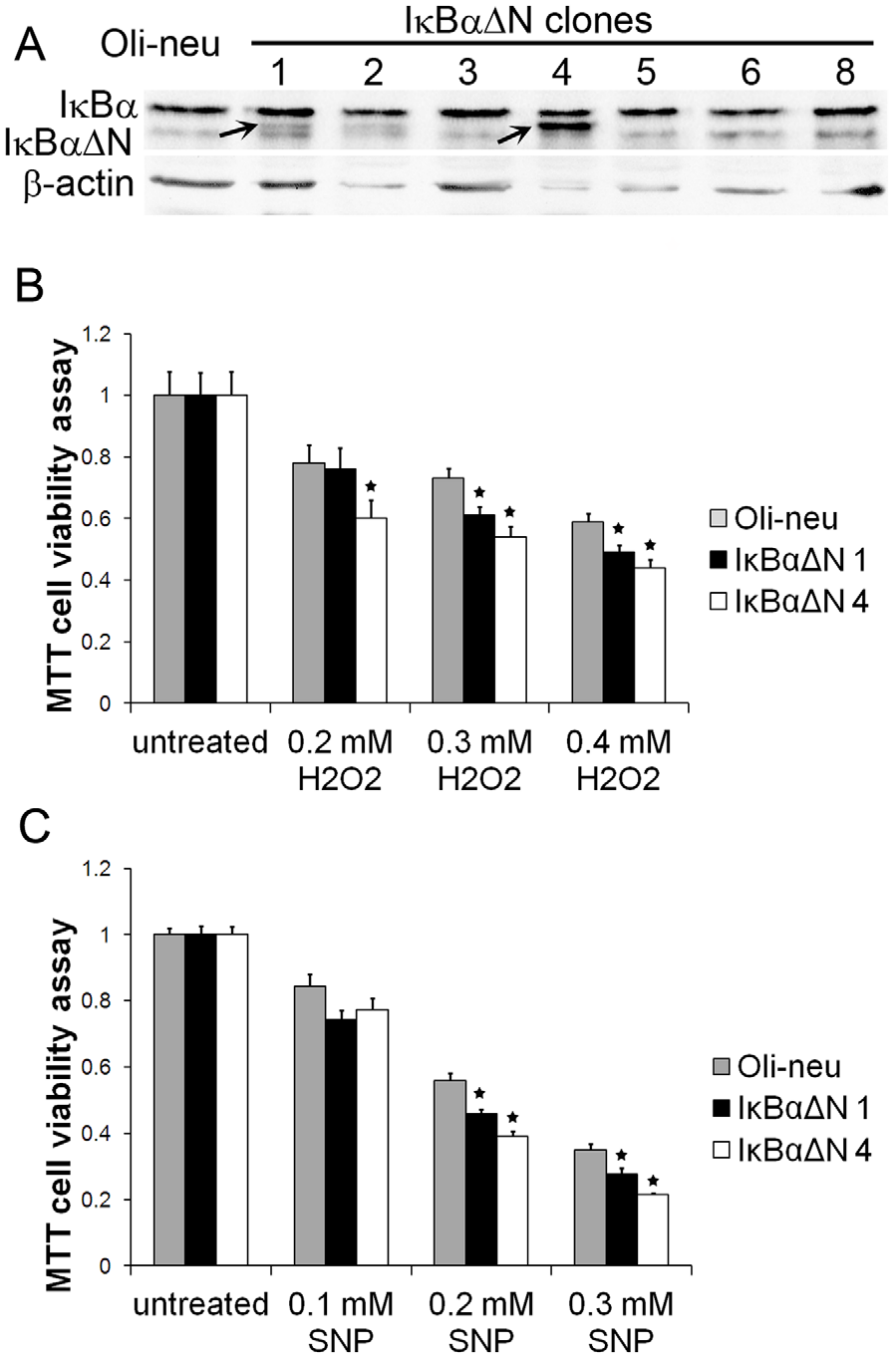
Enforced expression of IκBαΔN rendered Oli-neu cells sensitive to the cytotoxicity of reactive oxygen species and reactive nitrogen species. **A.** Oli-neu cells were transfected with the plasmid pcDNA3.1-IκBαΔN. We obtained several stably transfected cell lines that were resistant to hygromycin and expressed various levels of IκBαΔN. IκBαΔN 1 cells expressed moderate level of IκBαΔN (arrow) and IκBαΔN 4 cells expressed high level of IκBαΔN (arrow). **B.** The cells were treated with 0.2 mM, 0.3 mM, and 0.4 mM H_2_O_2_ for 24 hrs. MTT assay showed that the treatment with H_2_O_2_ reduced the number of Oli-neu cells in a dose-dependent manner and that enforced expression of IκBαΔN further reduced the cell numbers. **C.** The cells were treated with 0.1 mM, 0.2 mM, and 0.3 mM SNP for 24 hrs. MTT assay showed that the treatment with SNP reduced the number of Oli-neu cells in a dose-dependent manner and that enforced expression of IκBαΔN further reduced the cell numbers. The experiments were repeated at least three times, error bars represent standard deviation, asterisk *p*<0.05.

Next, we determined whether suppression of the NF-κB pathway rendered Oli-neu cells vulnerable to the cytotoxicity of IFN-γ. IκBαΔN, a deletion mutant lacking the N-terminal 36 amino acids of IκBα, is a dominant inhibitor of NF-κB signaling [25]. To suppress the activity of NF-κB, Oli-neu cells were transfected with a mammalian expression plasmid pcDNA3.1-IκBαΔN that contains the hygromycin resistance gene. We obtained several stably transfected cell lines that were resistant to hygromycin and expressed various levels of IκBαΔN (Figure 3A). Line 1 cells that expressed a moderate level of IκBαΔN (IκBαΔN 1) and line 4 cells that expressed a high level of IκBαΔN (IκBαΔN 4) were treated with 100 U/ml IFN-γ for 16 hrs. As expected, p65 and DAPI double labeling and confocal imaging analysis showed that enforced expression of IκBαΔN blocked IFN-γ-induced p65 nucleus translocation in IκBαΔN 1 and 4 cells (Figure 2A, B). EMSA analysis confirmed that enforced expression of IκBαΔN diminished IFN-γ-induced NF-κB activation (Figure 2C, D). Interestingly, MTT assay showed that IFN-γ treatment noticeably increased the magnitude of the reduction of cell numbers in IκBαΔN 1 and 4 cells compared to control Oli-neu cells (Figure 1A, the magnitude of the reduction in response to 150 U/ml IFN-γ treatment: 32%±1.7% in Oli-neu cells vs 43%±3.1% in IκBαΔN 1 cells and 50%±3.4% in IκBαΔN 4 cells). Caspase-3 activity assay also showed that IFN-γ treatment significantly increased caspase-3 activity in IκBαΔN 1 and 4 cells (Figure 1B). Moreover, active caspase-3 and DAPI double labeling showed that IFN-γ treatment significantly increased the number of apoptotic cells in IκBαΔN 1 and 4 cells, which were active caspase-3 positive and had condensed nuclei (Figure 1C, D). Additionally, we found that both caspase-3 activity and apoptotic cell numbers were significantly increased in the untreated IκBαΔN 1 and 4 cells compared to the untreated control Oli-neu cells (Figure 1B, D), which likely reflects that NF-κB is involved in supporting Oli-neu cell survival in the normal condition. Nevertheless, BrdU cell proliferation assay showed that suppression of the NF-κB pathway did not significantly alter the sensitivity of Oli-neu cells to the anti-proliferative effects of IFN-γ (Fig. 1E, the magnitude of the reduction of cell proliferation: 19%±4.85% in Oli-neu cells vs 23%±7.8% in IκBαΔN 1 cells and 24%±7.7% in IκBαΔN 4 cells). Collectively, our data show that NF-κB activation induced by IFN-γ protects Oli-neu cells against the cytotoxicity of this cytokine.

It has been shown that oligodendroglial lineage cells are susceptible to reactive oxygen species and reactive nitrogen species [26], [27], [28]. In accordance with these previous studies, MTT assay showed that the treatment with H_2_O_2_ reduced the number of Oli-neu cells in a dose-dependent manner (Figure 3B). Similarly, we found that the treatment with a NO donor SNP reduced the number of Oli-neu cells in a dose-dependent manner (Figure 3C). To determine the contribution of the NF-κB pathway in the response of Oli-neu cells to reactive oxygen species and reactive nitrogen species, IκBαΔN 1 and 4 cells were treated with H_2_O_2_ and SNP for 24 hrs, respectively. Interestingly, MTT assay showed that suppression of the NF-κB pathway made Oli-neu cells sensitive to the cytotoxicity of H_2_O_2_ (Figure 3B, the magnitude of the reduction of cell viability in response to 0.3 mM H_2_O_2_ treatment: 27%±4.2% in Oli-neu cells vs 39%±2.9% in IκBαΔN 1 cells and 46%±3.4% in IκBαΔN 4 cells) and SNP (Figure 3C, the magnitude of the reduction of cell viability in response to 0.2 mM SNP treatment: 44%±2.3% in Oli-neu cells vs 55%±1.3% in IκBαΔN 1 cells and 61%±1.7% in IκBαΔN 4 cells), respectively. These data further support that the NF-κB pathway promotes Oli-neu cell survival.

### PERK was Required for NF-κB Activation in Response to IFN-γ

**Figure 4:**
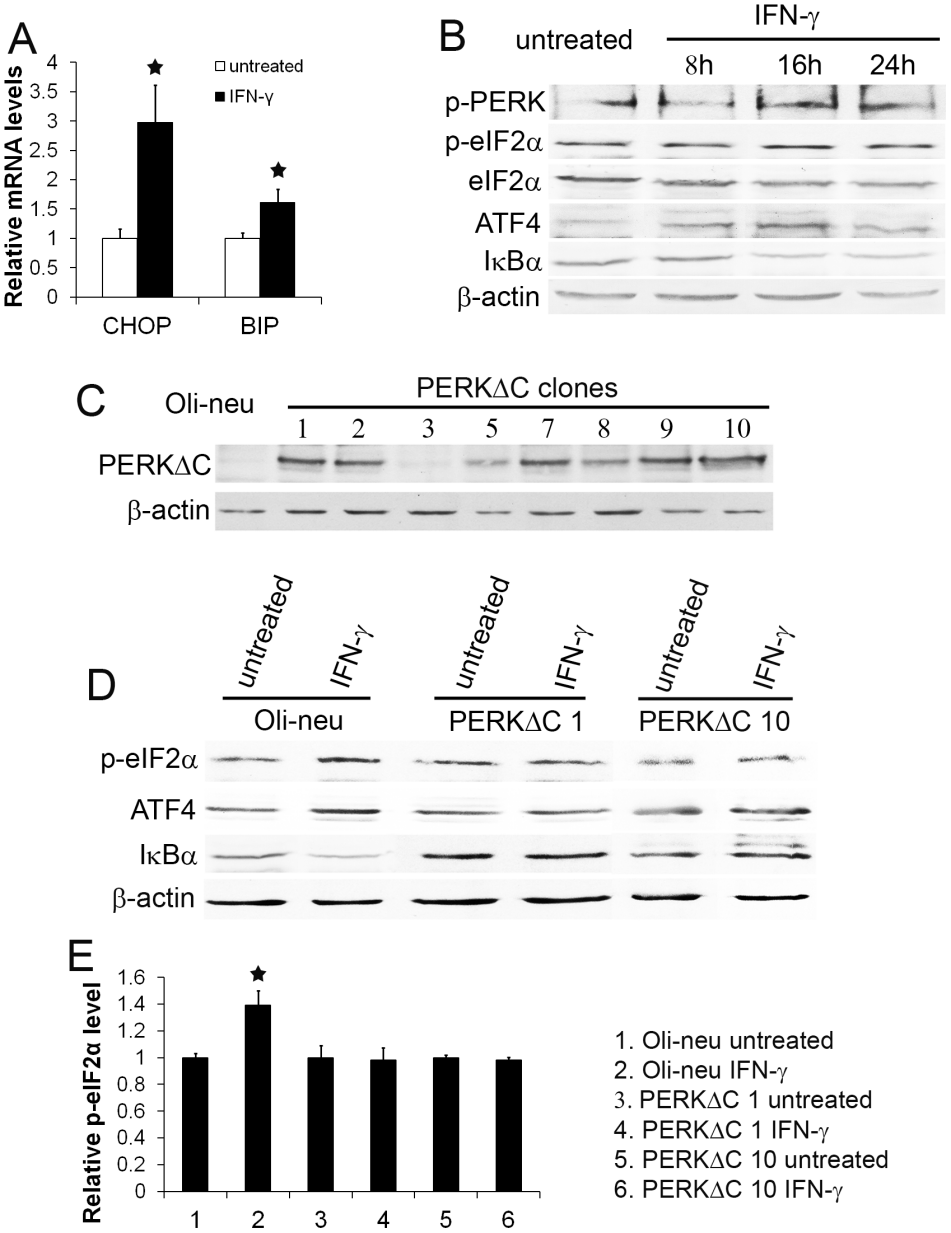
Enforced expression of PERKΔC diminished IFN-γ-induced activation of PERK signaling. **A.** Oli-neu cells were treated with 100 U/ml IFN-γ for 24 hrs. Real-time PCR analysis showed that IFN-γ treatment significantly increased the expression of CHOP and BIP in the cells. **B.** The cells were treated with 100 U/ml IFN-γ for 8 hrs, 16 hrs, or 24 hrs. Western blot analysis showed that the levels of p-PERK, p-eIF2α, and ATF4 in Oli-neu cells treated with 100 U/ml IFN-γ for 16 hrs were elevated compared to the untreated cells. Moreover, IFN-γ reduced the level of IκBα in Oli-neu cells after 16 hrs of treatment. **C.** Oli-neu cells were transfected with pBabe-PERKΔC vector encoding Myc epitope-tagged PERKΔC. Immunoblotting for Myc showed that stably transfected cell lines expressed various levels of PERKΔC. PERKΔC 1 and 10 cells expressed high level of PERKΔC. **D.** The cells were treated with 100 U/ml IFN-γ for 16 hrs. Western blot analysis showed that enforced expression of PERKΔC blocked IFN-γ-induced eIF2α phosphorylation and ATF4 upregulation in PERKΔC 1 and 10 cells. Western blot analysis also showed that enforced expression of PERKΔC diminished IFN-γ-induced reduction of IκBα level in PERKΔC 1 and 10 cells. **E.** Densitometry analysis of western blot results showed that IFN-γ treatment significantly increased the level of p-eIF2α in Oli-neu cells, but did not affect p-eIF2α level in PERKΔC 1 and 10 cells. The experiments were repeated at least three times, error bars represent standard deviation, asterisk *p*<0.05.

During ER stress, PERK activation stimulates the expression of numerous stress-induced cytoprotective genes by promoting the translation of the cytosolic transcription factor ATF4 [13], [14]. Activation of the PERK-eIF2α pathway also activates the NF-κB pathway through repression of IκBα translation [15], [16]. We have shown that IFN-γ induces ER stress in oligodendroglial lineage cells using primary cell cultures and animal models [3], [17], [18]. Interestingly, real-time PCR analysis showed that the levels of the UPR markers CHOP and BIP were significantly increased in Oli-neu cells treated with 100 U/ml IFN-γ for 24 hrs compared to the untreated cells (Figure 4A). Moreover, western blot analysis showed that the levels of p-PERK, p-eIF2α, and ATF4 in Oli-neu cells treated with 100 U/ml IFN-γ for 16 hrs were elevated compared to the untreated cells (Figure 4B, D, E). Additionally, we found that IFN-γ treatment reduced the level of IκBα in Oli-neu cells after 16 hrs of treatment (Figure 4B, D). These data demonstrate that IFN-γ activates the PERK pathway in Oli-neu cells.

**Figure 5:**
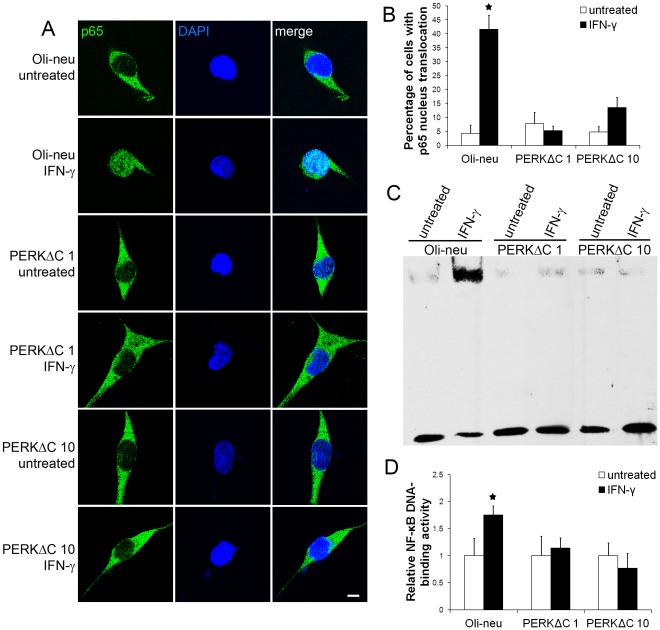
Enforced expression of PERKΔC impaired IFN-γ-induced NF-κB activation. **A, B.** The cells were treated with 100 U/ml IFN-γ for 16 hrs. p65 and DAPI double labeling and confocal imaging analysis showed that enforced expression of PERKΔC diminished IFN-γ-induced p65 nucleus translocation in PERKΔC 1 and 10 cells. **C,** **D.** The cells were treated with 100 U/ml IFN-γ for 16 hrs. EMSA analysis showed that enforced expression of PERKΔC blocked IFN-γ-induced increase in NF-κB DNA-binding activity in PERKΔC 1 and 10 cells. The experiments were repeated at least three times, error bars represent standard deviation, asterisk *p*<0.05, scale bar = 20 µm.

We further examined the involvement of PERK signaling in IFN-γ-induced NF-κB activation. PERKΔC, a kinase-defective PERK lacking the C-terminal kinase domain, is a dominant inhibitor of PERK signaling [20]. Oli-neu cells were transfected with pBabe-PERKΔC vector encoding Myc epitope-tagged PERKΔC [20]. We obtained several stably transfected cell lines that were resistant to puromycin. Immunoblotting for Myc showed that stably transfected cell lines expressed various levels of PERKΔC (Figure 4C). Lines 1 and 10 cells (PERKΔC 1 and 10) that expressed a high level of PERKΔC were treated with 100 U/ml IFN-γ for 16 hrs. As expected, western blot analysis showed that enforced expression of PERKΔC blocked IFN-γ-induced eIF2α phosphorylation and ATF4 upregulation in PERKΔC 1 and 10 cells (Figure 4D, E). Western blot analysis also showed that enforced expression of PERKΔC diminished the reduction of IκBα level in Oli-neu cells in response to IFN-γ. These data demonstrate that enforced expression of PERKΔC blocks activation of PERK signaling in Oli-neu cells in response to IFN-γ. Moreover, p65 and DAPI double labeling and confocal imaging analysis showed that enforced expression of PERKΔC diminished IFN-γ-induced p65 nucleus translocation in PERKΔC 1 and 10 cells (Figure 5A, B). Importantly, EMSA analysis showed that enforced expression of PERKΔC blocked NF-κB activation in PERKΔC 1 and 10 cells in response to IFN-γ (Figure 5C, D). Collectively, these data demonstrate that IFN-γ activates NF-κB in Oli-neu cells through PERK signaling.

**Figure 6:**
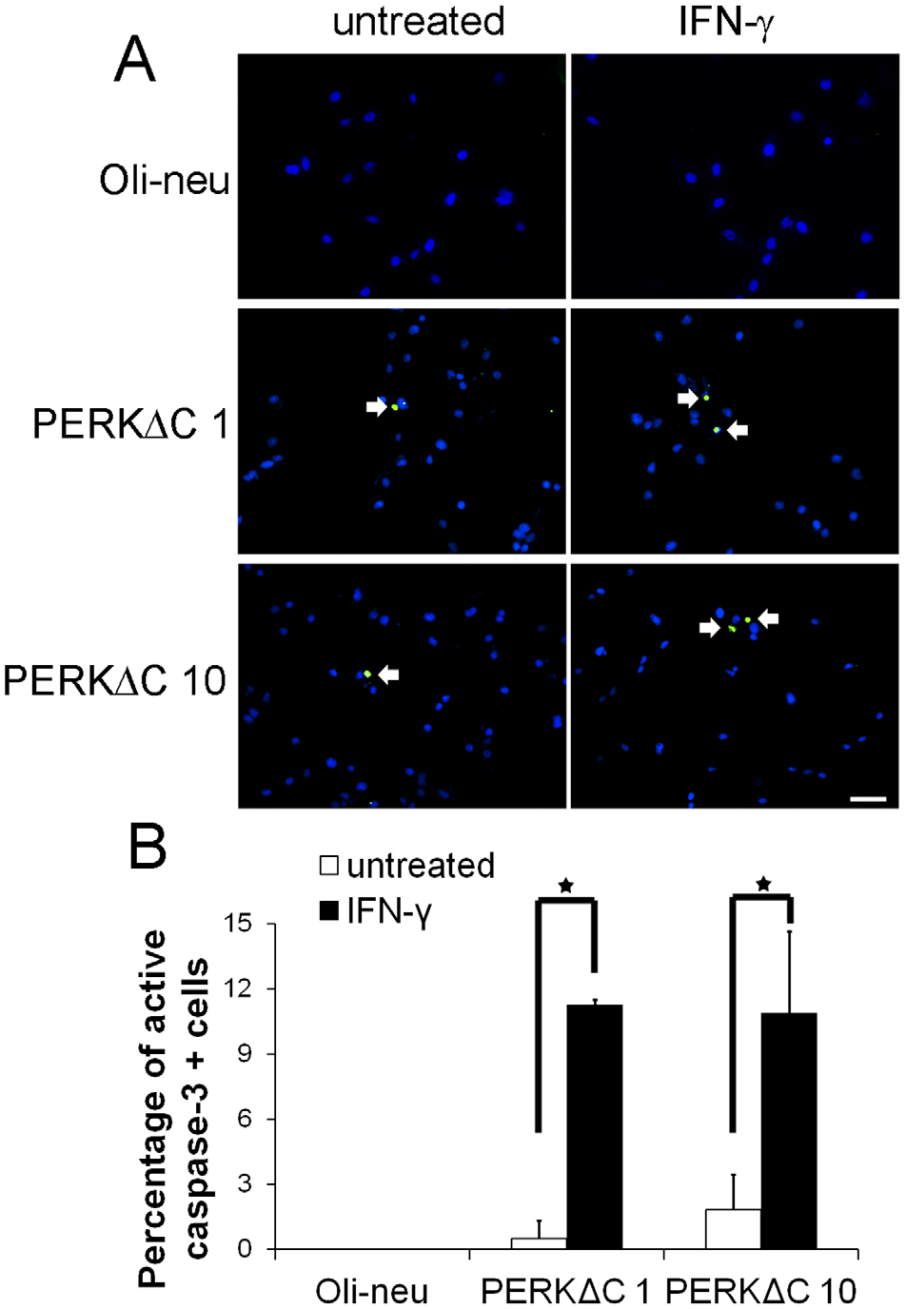
Enforced expression of PERKΔC made Oli-neu cells susceptible to the cytotoxicity of IFN-γ. **A,** **B.** The cells were treated with 100 U/ml IFN-γ for 24 hrs. Active caspase-3 and DAPI double labeling showed that IFN-γ treatment did not cause Oli-neu cell apoptosis, but significantly increased the number of apoptotic cells in PERKΔC 1 and 10 cells. The experiments were repeated at least three times, error bars represent standard deviation, asterisk *p*<0.05, scale bar = 100 µm.

It is well documented that PERK signaling is essential for cell survival during ER stress [13], [14]. Moreover, it has been shown that activation of PERK signaling protects oligodendrocytes against the cytotoxicity of IFN-γ [17], [18], [21]. To determine whether enforced expression of PERKΔC affects the viability of Oli-neu cells in response to IFN-γ, we performed active caspase-3 and DAPI double labeling. As mentioned above, we found that IFN-γ treatment did not cause Oli-neu cell apoptosis (Figure 1C, 1D, and 6). Nevertheless, active caspase-3 and DAPI double labeling showed that IFN-γ treatment significantly increased the number of apoptotic cells in PERKΔC 1 and 10 cells (Figure 6), which were active caspase-3 positive and had condensed nuclei. Taken together, these data demonstrate that blockage of PERK signaling makes Oli-neu cells susceptible to the cytotoxicity of IFN-γ, which is consistent with our previous studies [17], [18], [21].

### PERK Signaling Contributed to IFN-γ-induced NF-κB Activation in Oligodendrocytes *in vivo*

We have generated transgenic mice that allow for the temporally controlled delivery of IFN-γ to the CNS using the tetracycline-controllable system [29]. Using the mouse model, we have found that the presence of IFN-γ in the CNS during development results in myelinating oligodendrocyte death and activation of the PERK-eIF2α pathway in the cells [17]. We have also found that PERK deficiency impairs the activity of the PERK-eIF2α pathway in myelinating oligodendrocytes in the CNS of IFN-γ-expressing mice and exacerbates IFN-γ-induced oligodendrocyte death [17]. Moreover, our recent study showed that inactivation of the gene encoding GADD34, a stress-inducible regulatory subunit of a phosphatase complex that dephosphorylates eIF2α, enhanced the activity of the PERK-eIF2α pathway in myelinating oligodendrocytes in young, developing mice that express IFN-γ in the CNS and promoted the cell survival in response to IFN-γ [21].

**Figure 7:**
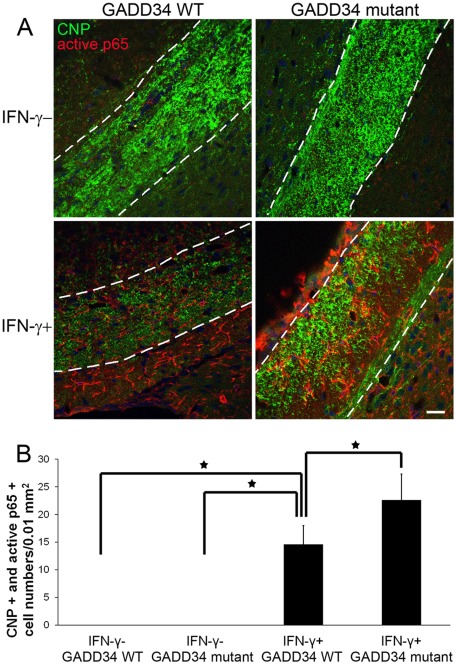
GADD34 inactivation increased IFN-γ-induced NF-κB activation in oligodendrocytes in IFN-γ-expressing transgenic mice. **A, B.** CNP and active p65 double immunostaining showed that the immunoreactivity of active p65 was undetectable in oligodendrocytes in the corpus callosum in control IFNγ ^ GADD34 WT mice and IFNγ ^ GADD34 mutant mice. The immunoreactivity of active p65 became detectable in a number of oligodendrocytes in the corpus callosum of IFNγ+GADD34 WT mice, and the number of active p65 positive oligodendrocytes was further increased in the corpus callosum of IFNγ+GADD34 mutant mice. The experiments were repeated at least three times, error bars represent standard deviation, asterisk *p*<0.05, scale bar = 20 µm.

To determine whether the PERK-eIF2α pathway was involved in IFN-γ-induced NF-κB activation in oligodendrocytes in immune-mediated demyelinating diseases, we exploited IFN-γ-expressing transgenic mice and *GADD34* mutant mice that have inactive GADD34 gene. *GFAP/tTA* mice and *TRE/IFN-γ* mice were mated with *GADD34* mutant mice, and the resulting progeny were intercrossed to obtain *GFAP/tTA; TRE/IFN-γ; GADD34* WT mice as well as *GFAP/tTA; TRE/IFN-γ; GADD34* mutant mice. The expression of the IFN-γ transgene was repressed in *GFAP/tTA; TRE/IFN-γ; GADD34* WT mice and *GFAP/tTA; TRE/IFN-γ; GADD34* mutant mice treated with doxycycline solution from conception (IFNγ ^ GADD34 WT mice and IFNγ ^ GADD34 mutant mice). The levels of IFN-γ became detectable around postnatal day 10 in the CNS of these mice that were released from doxycycline solution at embryonic day 14 (IFNγ+GADD34 WT mice and IFNγ+GADD34 mutant mice). In agreement with a previous study [30], CNP and active p65 double immunostaining showed that the immunoreactivity of active p65 was undetectable in oligodendrocytes in the corpus callosum in 21-day-old control IFNγ ^ GADD34 WT mice and IFNγ ^ GADD34 mutant mice (Figure 7). Importantly, we found that immunoreactivity of active p65 became detectable in a number of oligodendrocytes in the corpus callosum in 21-day-old IFNγ+GADD34 WT mice, and that the number of active p65 positive oligodendrocytes was further increased in the corpus callosum of IFNγ+GADD34 mutant mice. These results likely reflect that enhanced activation of the PERK-eIF2α pathway via GADD34 inactivation leads to elevated activation of the NF-κB pathway in oligodendrocytes in IFN-γ-expressing transgenic mice. Our data suggest that IFN-γ can activate NF-κB in oligodendrocytes in immune-mediated demyelinating diseases, and the PERK-eIF2α pathway contributes to IFN-γ-induced NF-κB activation. Additionally, these findings indicate that the beneficial effects of PERK signaling on oligodendrocytes in IFN-γ-expressing mice are associated with NF-κB activation.

## Discussion

IFN-γ is considered to be a key cytokine involved in the pathogenesis of immune-mediated demyelinating diseases [2], [5]. Several lines of evidence have suggested that the NF-κB pathway plays an important role in immune-mediated demyelinating diseases [7], [8], [9], [31], [32]. Interestingly, recent studies have shown that some biological effects of IFN-γ are elicited through activation of the NF-κB pathway [11], [12]. Our previous studies have shown that the effects of IFN-γ on oligodendrocytes in immune-mediated demyelinating diseases are mediated, at least in part, by the UPR [3], [17], [18]. Moreover, it has been shown that activation of the PERK branch of the UPR activates the NF-κB pathway in ER-stressed cells [15], [16]. Therefore, in this study, we determined the potential role of the NF-κB pathway in the effects of IFN-γ on oligodendrocytes and the molecular mechanism responsible for IFN-γ-induced NF-κB activation. First, using oligodendroglial cell line Oli-neu, we found that IFN-γ was capable of acting on the cells to activate the NF-κB pathway and that NF-κB activation was essential to protect Oli-neu cells against IFN-γ-induced apoptosis. Second, we showed that IFN-γ-induced NF-κB activation correlated with activation of PERK signaling and was abrogated in Oli-neu cells with enforced expression of PERKΔC, a dominant inhibitor of PERK signaling. Third, we showed that IFN-γ induced NF-κB activation in oligodendrocytes in transgenic mice that ectopically express IFN-γ in the CNS. Finally, we found that inactivation of the GADD34 gene enhanced IFN-γ-induced activation of the PERK-eIF2α pathway in oligodendrocytes in IFN-γ-expressing transgenic mice, resulting in increased NF-κB activation. Collectively, these data provide compelling evidence that the UPR induced by IFN-γ represents one mechanism by which IFN-γ triggers NF-κB activation in oligodendrocytes in immune-mediated demyelinating diseases.

It remains unclear how IFN-γ causes ER stress in oligodendrocytes. IFN-γ enhances the production of hundreds of distinct proteins in cells, including oligodendrocytes [33], [34]. It is possible that enhanced production of proteins leads to an overload of the capacity of the ER, resulting in ER stress. Many aspects of IFN-γ biology are exerted through the Janus kinases (JAKs) - signal transducer and activator of transcription 1 (STAT1) pathway [12], [35]. Recently, we have demonstrated that the presence of IFN-γ in the CNS activates the UPR in oligodendrocytes through the JAK-STAT1 pathway [36]. Activation of the PERK branch of the UPR adapts cells to the ER-stressed conditions by inhibiting global protein biosynthesis [13], [14]. Interestingly, it has been demonstrated that inhibition of IκBα biosynthesis by the PERK-eIF2α pathway leads to NF-κB activation [15], [16]. Herein, we showed that IFN-γ-induced NF-κB activation in Oli-neu cells correlated with the reduction of IκBα level. Importantly, we showed that enforced expression of PERKΔC diminished IFN-γ-induced reduction of IκBα level and NF-κB activation in Oli-neu cells. Taken together, our findings suggest that IFN-γ induces NF-κB activation by activating PERK signaling through the JAK-STAT1 pathway. On the other hand, recent studies suggest that multiple mechanisms are involved in IFN-γ-induced NF-κB activation. A study showed that IFN-γ activated the NF-κB pathway through a STAT1-independent pathway [37]. A recent report showed that IFN-γ activated IκB kinase β (IKK-β)-dependent NF-κB signaling through the JAK-STAT1 pathway [11]. Collectively, these data raise the possibility that the mechanisms responsible for IFN-γ-induced NF-κB activation are determined by the cell types. Additionally, this study does not rule out the possibility that other mechanisms are involved in IFN-γ-induced NF-κB activation in oligodendrocytes in immune-mediated demyelinating diseases. We have demonstrated that the presence of IFN-γ in the CNS increases the expression of tumor necrosis factor-α (TNF-α), a well characterized NF-κB inducer [21], [36]. An alternative, but not mutually exclusive, possibility is that IFN-γ activates NF-κB in oligodendrocytes in immune-mediated demyelinating diseases through the induction of TNF-α.

Oligodendrocytes are considered to be the target of immune attacks in immune-mediated demyelinating diseases [38]. Recent studies suggest that oligodendrocyte death contributes significantly to the development of immune-mediated demyelinating diseases [39], [40]. Moreover, oligodendrocyte regeneration and subsequent remyelination are thought to be necessary to restore neurological function in MS patients and EAE animals [41]. Several lines of evidence have suggested that the effects of IFN-γ on oligodendrocytes are dependent on the differentiation stages of the cells [5], [23], [24]. Our previous study has shown that IFN-γ protects mature oligodendrocytes in adult animals against immune attacks in EAE mice [3]. Moreover, we have demonstrated that the beneficial effects of IFN-γ on mature oligodendrocytes are associated with modest ER stress and that PERK deficiency diminishes the protective effects [3]. In contrast, we have shown that IFN-γ causes myelinating oligodendrocyte death in young, developing mice and remyelinating oligodendrocyte death in EAE demyelinated lesions [17], [18], [21]. We have also demonstrated that the detrimental effects of IFN-γ on (re)myelinating oligodendrocytes are associated with severe ER stress and that PERK deficiency makes (re)myelinating oligodendrocytes more sensitive to IFN-γ [17], [18]. On the other hand, it has been shown that IFN-γ influences the proliferation of oligodendrocyte precursors, but does not affect the viability of oligodendrocyte precursors [23], [24]. In agreement with these studies, we showed here that IFN-γ suppressed the proliferation of Oli-neu cells, an oligodendrocyte precursor cell line, but had no effect on the cell viability. Interestingly, we found that IFN-γ induced modest ER stress in Oli-neu cells and that blockage of PERK signaling resulted in the death of Oli-neu cells in response to IFN-γ. Collectively, these data indicate that activation of PERK signaling induced by IFN-γ is exclusively beneficial to oligodendrocytes in immune-mediated demyelinating diseases, in spite of double-edged sword effects of IFN-γ on the cells.

It is generally believed that inflammatory mediators, including immune cytokines, reactive oxygen species, and reactive nitrogen species, contribute to oligodendrocyte death in immune-mediated demyelinating diseases [27], [38]. Several studies have shown that NF-κB activation promotes immortalized oligodendrocyte survival in response to inflammatory mediators [26], [42], [43]. We showed here that NF-κB not only supported Oli-neu cell survival under the normal condition but also promoted Oli-neu cell survival in response to IFN-γ, reactive oxygen species, and reactive nitrogen species. Our data provide additional evidence that NF-κB activation is beneficial to oligodendrocyte survival in immune-mediated demyelinating diseases. We also showed that IFN-γ induced NF-κB activation in Oli-neu cells through PERK signaling. On the other hand, our previous study has shown that GADD34 inactivation enhances the activity of PERK signaling in myelinating oligodendrocytes and promotes the cell survival in young, developing mice that express IFN-γ in the CNS [21]. Importantly, in this study, we showed that the GADD34 inactivation enhanced IFN-γ-induced NF-κB activation in oligodendrocytes in the mice. These findings indicate that NF-κB activation contributes to cytoprotective effects of PERK signaling on oligodendrocytes in IFN-γ-expressing mice. Additionally, it has been demonstrated that both the PERK pathway and the NF-κB pathway are activated in oligodendrocytes in immune-mediated demyelinating diseases [3], [8], [44]. Taken together, these data raise the possibility that the cytoprotective effects of IFN-γ-induced PERK activation on oligodendrocytes in immune-mediated demyelinating diseases are mediated by the NF-κB pathway. In contrast, a recent study showed that oligodendrocyte-restricted deletion of IKKβ had no noticeable effects on oligodendrocyte viability in mice with EAE [30]. Both IKKβ-dependent and IKKβ-independent pathways are involved in NF-κB activation in cells [45]. Additionally, many signaling pathways have been shown to participate in manipulating the activity of the NF-κB pathway [46]. Therefore, the dispensable role of IKKβ deletion in oligodendrocyte viability likely reflects that IKKβ deletion alone does not significantly decrease the activity of the NF-κB pathway in oligodendrocytes in EAE mice. Clearly, the precise role of the NF-κB pathway in oligodendrocytes in immune-mediated demyelinating diseases warrants further investigation. A transgenic mouse model that allows for the temporally controlled expression of IκBαΔN, a super-suppressor of NF-κB, exclusively in oligodendrocytes could be an ideal model to address this important open question.

In summary, we have demonstrated that the PERK branch of the UPR contributes to IFN-γ-induced NF-κB activation in oligodendrocytes. Moreover, our findings indicate that NF-κB activation is beneficial to oligodendrocytes. As such, this study reveals a novel mechanism by which IFN-γ activates the NF-κB pathway. Additionally, the results presented in this study suggest that NF-κB activation by IFN-γ represents one mechanism by which IFN-γ exerts its effects on oligodendrocytes in immune-mediated demyelinating diseases.
